# Vexed causal inferences in nutritional epidemiology—call for genetic help

**DOI:** 10.1093/ije/dyab152

**Published:** 2021-08-13

**Authors:** Pauli Ohukainen, Jyrki K Virtanen, Mika Ala-Korpela

**Affiliations:** 1 Computational Medicine, Faculty of Medicine, University of Oulu and Biocenter Oulu, Oulu, Finland; 2 Center for Life Course Health Research, University of Oulu, Oulu, Finland; 3 Institute of Public Health and Clinical Nutrition, University of Eastern Finland, Kuopio, Finland; 4 NMR Metabolomics Laboratory, School of Pharmacy, University of Eastern Finland, Kuopio, Finland

Nutritional epidemiology is criticized for its inability to provide plausible information on the causal effects of diet on health and disease outcomes, a key to both scientific understanding and guiding public policies.^1^^,^^2^ Although most public dietary guidelines globally have arrived at similar conclusions, they all continue to rely strongly on observational studies.^3^ It is well known in epidemiology that observational study settings produce the lowest-quality data with perceivable but also unidentifiable confounding and with very limited, if any, opportunities to assess direct causality. However, we would hope for the same reliability for nutritional recommendations as for pharmacological treatments—only substances known to have causal effects should be recommended and used.^4^ Thus, randomized controlled trials (RCTs) might be seen as a favourable strategy to produce reliable causal evidence also in nutritional research.^1^ However, RCTs attempting to study the effects of individual foods or nutrients face severe obstacles. For example, a diet always consists of multifactorial and synergistic components, so it is unrealistic for nutritional RCTs to address all the potentially meaningful components of diets, and it may also take years or even decades to have noticeable dietary effects on relevant health outcomes. Low compliance and high dropout rates are also common in nutrition studies if participants are asked to change their typical diets for more than a few months. Blinding, one of the cornerstones of RCTs, is often impossible if the study requires changes in dietary intakes that cannot be accomplished by supplementation. There is also rarely a placebo group with zero intake of a certain nutrient.

Vitamins provide a classical example of the challenges detailed above.^5^ Multiple observational outcome associations, like for plasma vitamin C (vitC), reflect extensive confounding and, even after adjustment for a range of confounders, residual confounding cannot be ruled out. Measurement errors in the applied confounders and failure to include all relevant confounding factors, e.g. socioeconomic and behavioural, from across the life course will be involved and explain the contradicting null results from RCTs.^5^ In recent large-scale Mendelian randomization studies, Dutch researchers looked at the potential causal effects of diet-derived circulating antioxidants (vitamins E and C, retinol, beta-carotene and lycopene) on the risk of coronary heart disease^6^ and stroke.^7^ The results in both studies do not support a protective effect of dietary-derived antioxidant levels on the outcome risk. Thus, it is unlikely that antioxidant supplementation at the population level would be beneficial for the prevention of coronary heart disease or stroke. The total study population in these analyses was extensive, 768 121 individuals with 93 230 cases and 1 065 119 individuals with 77 612 cases for the coronary heart disease^6^ and stroke,^7^ respectively.

The above-mentioned limitations of both observational nutritional epidemiology and related RCTs would call for additional approaches to complement nutritional research to facilitate causal inference. Genetic instrumentation has been suggested as a tool for aiding causal inference in nutritional epidemiology.^2^ In this Opinion, we tackle recent ideas and applications of Mendelian randomization analysis in big data and elucidate the appeal of these new scientific approaches in genetic epidemiology.

## Dietary habits and Mendelian randomization analysis

A recent work by Cole *et al.*,^8^ focusing on the genetic background of dietary habits, presents an interesting approach. The authors performed a genome-wide association study (GWAS) on 85 single food intake and related dietary patterns from food frequency questionnaires in the UK Biobank. Over 800 associated genetic loci were identified, allowing the set-up of specific genetic instruments and the application of Mendelian randomization analysis to assess potential causal effects on various health outcomes. The authors reported, for example, that a genetically associated dietary pattern driven by wholemeal vs white bread consumption would be causally influenced by factors correlated with education but would not be causal for coronary artery disease or type 2 diabetes. In fact, instead of demonstrating a causal effect from diet to disease, the findings were interpreted to suggest a reverse causal relationship between coronary artery disease and diet—maybe reflecting a potential behavioural change towards believed healthier food choices due to a heart disease diagnosis. This is analogous to other examples of genetic associations being subject to reverse causality. For example, even though a genetic risk score for coronary heart disease is associated with statin therapy, the true causal pathway is that the disease causes an individual to be prescribed statins, not the other way around.

**Figure 1:**
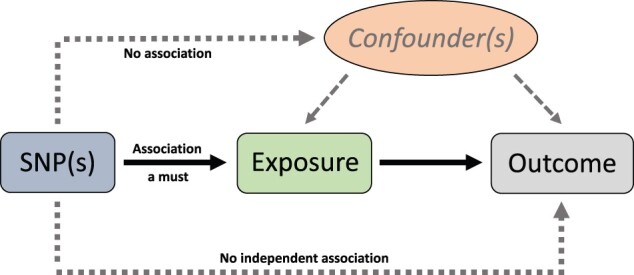
The fundamental principles and prerequisites for univariable Mendelian randomization analysis to estimate causal relationships. There are three key principles in this instrumental variable analysis. The genetic variant (either in isolation or in combination with other variants) must associate with the exposure but must not associate with either known or unknown confounders, and there should be no pathway from the genetic variant(s) to the outcome which does not include the exposure of interest. In two-sample Mendelian randomization analysis, the single nucleotide polymorphism (SNP)-to-exposure estimate is obtained from a dataset separate from that of the SNP-to-outcome estimate. This allows the use of the best existing genome-wide association study (GWAS) for both the exposure (e.g. a circulating biomarker) and the outcome (e.g. coronary heart disease) in rather common situations where a single appropriate dataset is not available. This is a schematic representation and should not be interpreted as a formal directed acyclic graph.

Mendelian randomization is a form of instrumental variable analysis used to assess causality of exposures using genetic data and it has become increasingly popular in epidemiology over the past decade.^4^^,^^9^ A clear distinction should be made between Mendelian randomization and a GWAS, the former being based on the latter. This is also reflected in the interpretation of results: a GWAS provides genetic associations in a general sense but the Mendelian randomization framework assesses potential causal effects.^10^ The fundamental principles and prerequisites for univariable Mendelian randomization analysis are exemplified in Figure 1. The three axioms of Mendelian randomization analysis are: (i) the genetic variant(s) must associate with the exposure; but (ii) not with either known or unknown confounders; and (iii) there should be no pathway from the genetic variant(s) to the outcome which does not include the exposure of interest. In nutritional epidemiology, however, the interpretation of the genetic component of predominantly environmental traits, such as dietary intake, is complicated and prone to various caveats, as also emphasized by Cole *et al.*^8^ Dietary habits are highly correlated both with each other and with non-dietary traits, suggesting that any single dietary phenotype may represent a broader diet and lifestyle, for example, with confounding links to obesity and socioeconomic status. From a methodological point of view, inevitable genetic pleiotropy and weak instruments complicate the quest for robust findings. It should not be surprising that our preference, e.g. for eating a certain type of bread, can at best be weakly affected by genetics but is heavily dependent on cultural and socioeconomic determinants. Thus, genetic instrumentation and Mendelian randomization analyses might not provide much help in assessing the causality of intake of a single food or dietary pattern on outcomes in a univariable setting.^8^ However, there can be specific cases in which a genetic variant provides a good instrument for mimicking the dietary intake of a single nutrient. The observational^11^ and RCT evidence^12^ in relation to vitC has been complemented with a Mendelian randomization study.^13^ Variation in the solute carrier family 23 member 1 (*SLC23A1*) gene is robustly associated with circulating vitC levels,^14^ making it a rare but excellent instrumental variable for studying potential long-term causal effects of this nutrient.

The complex relationships between genetics, dietary exposures and potential causal pathways with health outcomes were recently elaborated by Pirastu *et al.*^15^ They developed a statistical genetics framework, involving multivariable Mendelian randomization analysis, to better account for confounding and collider bias in order to increase the robustness of causal analyses.^16^ They demonstrated that genetic associations with dietary traits are likely also affected by reverse causality, thus giving a potential explanation of the heterogeneity of genetic correlations and genome-wide associations in different populations. The authors noted that when considering the effects of foods on health, the genetic evidence would support the importance of dietary patterns rather than single foods or nutrients. This method has also recently been applied to study the potential causal associations of dietary patterns on over a hundred circulating biomarkers analysed by nuclear magnetic resonance (NMR) metabolomics.^17^ These Mendelian randomization analyses identified more than 400 potentially causal links between food and biomarkers with replication of some previous findings, e.g. increased oily fish consumption and higher circulating docosahexanoic acid (DHA) concentrations. Among new causal findings were various food effects on apolipoprotein B-containing lipoprotein particles.^17^

In another recent study using the UK Biobank data, Dashti *et al.* analysed the potential causal effects of morning-evening diurnal preferences on food intake.^18^ Their results suggest that a morning diurnal preference causes increased intake of higher-quality foods and decreased intake of lower-quality foods. These results may reflect temporality in the consumption of foods and thus warrant the assessment of diurnal preferences in public health and in relation to dietary advice.

## Genetic pleiotropy

It is important to note that genetic pleiotropy is an established and pervasive characteristic of the human genome. Consequently, we should not think as simply as ‘one gene, one function, one trait’, but be aware that many single genetic variants influence a plethora of different traits.^19^ A violation of the prerequisites for Mendelian randomization analyses is the so-called horizontal pleiotropy that refers to genetic variants influencing two traits via independent pathways. However, a genetic variant influencing other traits on the same pathway (so-called vertical pleiotropy) is not a direct violation of the axioms of Mendelian randomization analysis. The obstacle of (horizontal) pleiotropy has been tackled extensively in the literature and readers are referred to a recent review by Hemani *et al.*^20^ for further details.

The studies by Papadimitriou *et al*.^21^ and Burrows *et al*.^22^ are recent exemplars of how to control for genetic pleiotropy via extensive sensitivity analyses in nutritional Mendelian randomization applications. Papadimitriou *et al*. tackled the inconsistencies in observational associations between the consumption or circulating concentrations of micronutrients and breast cancer risk. Of the 11 micronutrients studied, higher concentrations of magnesium were causally related to the increased risk of breast cancer.^21^ Burrows *et al*. challenged the contradictory observational evidence on associations between folate and the risk of several common cancers. Their results gave little evidence that serum folate would be causal for pan-cancer or various site-specific cancers, suggesting that increasing levels of circulating folate is unlikely to lead to population-wide increase in cancer risk.^22^

## Mediating biomarkers and multivariable causal mediation analysis

**Figure 2:**
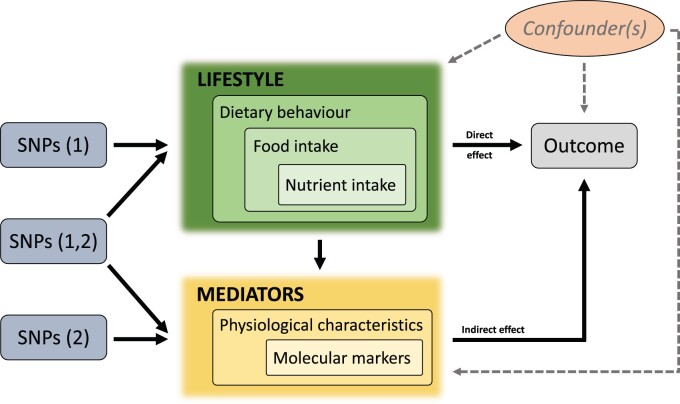
A general schematic illustration of multivariable Mendelian randomization analysis in a mediation scenario to assess direct causal effects of lifestyle-related dietary factors and their indirect causal effects as mediated by physiological and molecular exposures. Single nucleotide polymorphisms (SNPs) (1) refers to the genetic variants affecting the lifestyle-related dietary factors; SNPs (2) refers to the genetic variants affecting the physiological and molecular exposures; and SNPs (1,2) refers to the genetic variants affecting both exposures. Consequently, the causal effects estimated by univariable and multivariable Mendelian randomization analysis can differ. Univariable estimates represent the total causal effect of the exposure on the outcome, whereas multivariable estimates constitute the direct causal effect of each exposure on the outcome. This is a schematic representation of a common situation in nutritional epidemiology research and should not be interpreted as a formal directed acyclic graph.

In spite of the above-mentioned methodological complexities, and even some unfounded resistance expressed by some prominent researchers,^23^ we anticipate that new developments with multivariable Mendelian randomization (MVMR) analysis are of particular value in nutritional epidemiology. In MVMR, many scenarios are possible between exposures, mediators and an outcome.^24^ This approach also retains the benefits of using genetic instruments for causal inference, thus avoiding bias due to confounding while allowing for estimation of the different effects required for mediation analysis. As we demonstrate in Figure 2, in the assessment of the effects of various lifestyle-related dietary factors, we can consider several likely physiological and molecular mediators between the primary exposures and the outcome.^24^ Both direct and indirect causal associations are likely in many nutritional situations. Here we would like to focus on the potential of Mendelian randomization analyses to support nutritional epidemiology, particularly with respect to the robustness of the interpretations of potential causal consequences of dietary observations. Reliable Mendelian randomization analyses are already available for many biomarkers to aid causal interpretations in nutritional studies.^4^^,^^9^^,^^23^

Table 1 provides an exemplary list of markers affected by diet, related Mendelian randomization evidence and RCT data. For example, systolic blood pressure is known to be affected by diet^56^ and its causality for cardiovascular disease (CVD) is supported by both Mendelian randomization analyses and RCTs,^54^^,^^55^ endorsing it as a valid mediating biomarker in nutritional epidemiology. Conversely, although low selenium status was observationally associated with higher risk of prostate cancer,^39^ subsequent RCTs with selenium supplementation were discontinued due to increased risk,^41^ and the same outcome was later reported in a Mendelian randomization analysis.^40^ In general, it would be essential to realize that observational epidemiology cannot elucidate causality but, when Mendelian randomization analyses have been possible, they have usually produced consistent results with RCTs.^23^

**Table 1:** Selected examples of associations between diet-related biomarkers and various disease outcomes via observational study settings, Mendelian randomization (MR) analysis and randomized controlled trials (RCTs)

Diet or supplementation biomarker	Disease outcome	Observational evidence	Mendelian randomization evidence for causality	RCT evidence for causality	Interpretation
Vitamin D	Cardiovascular disease	Inverse association with cardiovascular mortality^25^	No causal relationship for coronary artery disease^26^	No benefit in a meta-analysis of 21 clinical trials^27^	MR analyses and RCTs are consistent: elevating serum vitamin D by supplementation unlikely to reduce cardiovascular mortality
	Colorectal cancer	Inverse association^28^	No causal relationship^29^	No reduction of risk by supplementation^30^^,^^31^	MR analyses and RCTs are consistent: elevating serum vitamin D by supplementation unlikely to reduce risk
	Breast cancer	Inverse^32^ or no association^33^	No causal relationship^29^	No reduction of risk by supplementation^34^	MR analyses and RCTs are consistent: vitamin D unlikely associated with risk; RCTs are contraindicated
	Prostate cancer	No association^33^	No causal relationship^29^	No reduction of risk by supplementation^31^	All studies are consistent: vitamin D unlikely associated with risk; RCTs are contraindicated
Homocysteine	Stroke	Positive association^35^	A causal relationship^36^	Meta-analysis of 25 trials of homocysteine lowering (with vitamin B)^37^ indicates a causal effect.	All information is consistent: lowering serum homocysteine likely to reduce risk of stroke
	Ischaemic heart disease	Positive association^35^	No causal relationship^38^	Meta-analysis of 10 placebo-controlled studies shows no benefit^38^	MR studies and RCTs are consistent: lowering serum homocysteine unlikely to reduce risk of ischaemic heart disease
Selenium	Prostate cancer	Toenail selenium inversely associated with risk^39^	A causal relationship for blood selenium^40^	Trial of selenium (and vitamin E) supplementation discontinued due to increased risk of prostate cancer^41^	MR analyses and RCTs are consistent: increasing selenium intake likely to increase risk
HbA1c	Cardiovascular disease	Positive association with higher risk of mortality and myocardial infarction in patients with coronary artery disease^42^	A causal relationship^43^	SGLT2i reduces HbA1c and CVD mortality in type 2 diabetic patients^44^	All information is consistent: lowering HbA1c likely to prevent CVD
Vitamin E	Coronary heart disease	Inverse association^45^	A causal relationship^46^	No benefit in high-risk individuals^10^	MR analyses and RCTs are consistent: No protective effect of supplementation and potential safety concerns
IL-6	Rheumatoid arthritis	Positive association^47^	An inverse causal relationship^48^	Inhibition of IL-6 receptor with tocolizumab reduces disease activity and improves function^49^	MR analyses and RCTs are consistent: inhibition of IL-6 pathway is beneficial
Calcium	Cardiovascular disease	Positive association^50^	A causal relationship^51^	Supplementation increases risk of vascular outcomes^52^	All information is consistent: serum calcium positively associated with risk of CVD
Blood pressure	Cardiovascular disease	Positive association^53^	A causal relationship^54^	Lowering blood pressure prevents cardiovascular disease^55^	All information is consistent: lowering blood pressure is beneficial for prevention and treatment of CVD
Vitamin C	Cardiovascular disease	Inverse association^11^	No causal relationship^13^	Supplementation does not reduce risk of cardiovascular disease^12^	MR analyses and RCTs are consistent: supplementing with vitamin C does not reduce CVD

Studies listed do not represent a chronological accumulation or the totality of a specific type of evidence. In most cases, RCT evidence precedes genetic evidence. There are also often multiple observational studies for the same exposure and outcome, but only one representative work is cited. A narrative reference tracking search strategy was used to identify examples where all three types of studies addressing the same exposure were available.

CVD, cardiovascular disease; HbA1c, glycated haemoglobin; IL-6, interleukin-6; SGLT2i, sodium-glucose cotransporter 2 inhibitor.

A particularly fruitful area of research may lie at an intersection of RCTs and Mendelian randomization analysis. Instead of attempting to stretch nutritional trial durations long enough to cover clinical endpoints, a more practical approach may be to first conduct a smaller and shorter RCT followed by Mendelian randomization. This ‘two-step randomization’ approach has been performed to investigate potential clinical benefits of lycopene supplementation on prostate cancer risk.^57^ First, a feasibility trial of lycopene supplementation^58^ was done to determine the systemic metabolic effects of the intervention. Circulating pyruvate concentrations were lowered and the long-term causal effects of this were then investigated in a Mendelian randomization framework, suggesting a lowered prostate cancer risk.^57^ The logic in this clinical interpretation relies on the assumption that the supplementation effects observed in the short-term intervention step would be sustained long term. On the other hand, the results from the Mendelian randomization with respect to the causal role of circulating pyruvate for prostate cancer are valid regardless, as they indicate the lifelong genetic effects of the metabolite in question.^4^^,^^9^

## Perils of hasty causal expectations—‘good cholesterol’ and vitamin D

Recent years have provided a cautionary exemplar of over-reliance on observational epidemiology, namely circulating high-density lipoprotein cholesterol (HDL-C), the so-called ‘good cholesterol’ for many years. Since the early days of cardiovascular research, low HDL-C has been seen as an unquestionable risk factor for CVD, but the situation has become intricate with modern genetics.^4^ Coherent observational findings led to a hypothesis that any intervention elevating circulating HDL-C would lower the risk of CVD. However, multiple clinical trials testing this hypothesis have either failed or demonstrated benefit unrelated to raising circulating HDL-C concentrations.^59^^,^^60^ Consistent with these null trial outcomes, various Mendelian randomization analyses have also indicated that HDL-C would not be causal for CVD.^61^ The latest evidence is also questioning the causality of apolipoprotein A-I (apoA-I), together with some of the functional features of HDL particles—a bad omen for the ongoing apoA-I infusion trials.^62^

In nutritional epidemiology, a story with similar features is unfolding around vitamin D (vitD). The observational evidence for the association between low circulating vitD and multiple disease outcomes is strong and long-lived. However, recent RCTs of vitD supplementation in the prevention of cancer^30^ and cardiovascular disease^31^ have led to null results (Table 1). Almost simultaneously, Mendelian randomization analyses have suggested that vitD would not be causal for schizophrenia, prostate cancer or bone mineral density but may be for ovarian cancer, multiple sclerosis and Alzheimer’s disease.^63^ RCTs of vitD supplementation are complicated by issues around the optimal dosing regimen, study duration, baseline vitD status and non-supplement sources of vitD. Ethical considerations are also paramount. For example, how long can we monitor vitD-deficient participants? Due to numerous difficulties in conducting an optimal nutritional trial, fundamental questions about causality can be excessively difficult to answer. Serum vitD is an example of an exposure that can have multiple upstream drivers, including diet. Nevertheless, despite the inherent difficulties in directly assessing the potential causal effects of dietary vitD, we can use serum vitD as an exposure in Mendelian randomization analyses. Even though population determinants of vitamin D status are numerous, circulating vitD is modified by supplementation and the well-characterized genetic regulation allows the build-up of an appropriate genetic instrument.^64^

## Bidirectional Mendelian randomization for vitamin D and multiple diseases

A recent genome-wide association study of vitD concentration in the UK Biobank (which identified 143 independent loci), together with bidirectional Mendelian randomization analyses, is an elegant exemplar of how the causal role of a biomarker can be extensively evaluated.^65^ After paying particular attention to accounting for genetic pleiotropy, an overall conclusion of these analyses was that there was no robust evidence that vitD would have causal effects on body mass index and multiple disease phenotypes. In addition, Revez *et al*.^65^ concluded that observational epidemiological links between vitD and psychiatric disorders mostly reflect confounding and/or reverse causation. In general, this recent work demonstrates the possibilities of Mendelian randomization and also suggests that many phenotypes do appear to have causal effects on vitD concentration, thereby emphasizing an important role of reverse causation for this particular biomarker and reminding us to be sceptical in interpreting evidence arising from observational epidemiological studies.

## Dietary fats and cardiovascular disease

The long-term nutritional research on the potential direct health effects of dietary fats is an exemplar of the vast difficulties in acquiring robust causal evidence. The early research suggested that the reduction of saturated fats, especially when replaced with polyunsaturated fats, would be beneficial for cardiometabolic health.^66^ Serum total cholesterol was suggested as a potential mediator of this effect, leading to the classic ‘diet-heart hypothesis’. Total cholesterol was eventually replaced by low-density lipoprotein cholesterol (LDL-C) but the hypothesis remained essentially the same. Controlled feeding studies in metabolic wards have established that consuming saturated fats leads to increased LDL-C concentrations,^67^ a finding also supported by mechanistic studies.^68^ Nevertheless, rigorous testing of this hypothesis has proved challenging and the present-day dietary guidelines have been questioned, for example, due to the lack of independent association between dietary saturated fat and CVD in more recent epidemiological studies^69^ and the lack of benefit when replacing saturated fats with omega-6 fatty acids in RCTs.^70^ Fundamental issues of confounding from the food matrix of individual foods as well as the totality of diet and varying effects of different species of saturated fatty acids also apply.

Recent observational findings from the large-scale Prospective Urban Rural Epidemiology study, with over 125 000 participants from 18 countries from five continents, have been interpreted to be at odds with current recommendations to reduce total fat and saturated fats.^71^^,^^72^ The results reveal that nutrients have varying effects on different circulating lipoprotein risk factors. The authors argue that predicting the net clinical effect based on considering only the effects of nutrient intake on LDL-C would not be reliable in projecting the effects of diet on CVD events or on total mortality. The situation is complicated by the fact that some lipid biomarkers are causal for cardiometabolic outcomes (LDL-C, apolipoprotein B and triglycerides)^73^ but, as discussed above, the recent genetic evidence strongly suggests, in contradiction to the observational associations, that HDL-C and apoA-I would not be causal.^61^^,^^62^ Within the caveats discussed above in relation to epidemiological studies as well as nutritional RCTs, it also needs to be kept in mind that whatever the effects of fats on plasma lipids, we would need to remain cautious in causal interpretations between fats and disease outcomes. Nevertheless, the most recent meta-analysis of RCT evidence does suggest that reducing saturated fat intake for at least 2 years causes a potentially important reduction in combined cardiovascular events.^74^ The controversy is therefore likely to remain and, alas, Mendelian randomization analysis cannot help in this case since there is no specific biomarker for saturated fat intake (serum saturated fatty acids are not a reliable proxy of dietary saturated fatty acids).

A partial solution to the above-mentioned instrument problem has been recently provided by a GWAS of relative macronutrient intake.^75^ Implementing these single nucleotide polymorphisms (SNPs) in a two-sample Mendelian randomization setting, Park *et al.* were able to assess the causal associations of relative macronutrient intake on the risk of chronic kidney disease (CKD).^76^ Their results suggested that, for a given level of total calorie intake, the composition of macronutrient intake would causally affect the risk of CKD. Thus, reducing relative fat intake and increasing relative protein intake may causally reduce the risk of CKD in the general population.

## Mediterranean diet and cardiovascular disease

The Mediterranean diet is generally seen favourable for cardiometabolic health. In a recent study, Li and co-workers developed a quantitative score for the adherence to a traditional Mediterranean diet and further linked the score to a systemic metabolic signature.^77^ Although the association between the Mediterranean diet score and the metabolic signature was weak, this approach allowed the application of Mendelian randomization analysis via the genetic instrumentation of the metabolic signature. The genetic component of the signature was inversely associated with risk of coronary heart disease and stroke. Several risk factors, including blood lipids, systolic blood pressure and diabetes, showed weak mediating effects in these Mendelian randomization analyses. In general, the quantification of habitual diet (with or without related biomarker signatures) may be a more realistic concept to assess causal pathways from diet to disease than focusing on individual foods and nutrients. Further genetic support for the favourable effects of the Mediterranean diet, high in vegetables, comes from a recent study by Park *et al*., suggesting that higher vegetable intake may be causally associated with better kidney function.^78^

## Better appreciation of causality—better quality nutritional epidemiology

Determining reliable and specific biomarkers for food intake is extremely challenging, if not impossible. It should also be noted that diet naturally has effects on multiple biomarkers and thus a single one would be highly unlikely to capture the physiological effects successfully. However, with Mendelian randomization analysis we now have a potentially powerful tool to assess causality of various biomarkers. Dietary behaviours and individual nutrients are most likely linked to several biological mediators in the human (patho)physiology. Although we may never know or be able to measure all of them, striving to identify causal mediators allows us to make stronger causal inferences for dietary exposures. If none are available or instrumental variable analyses disagree with the observational findings, wider exploration of alternative hypotheses, including methodological shortcomings related to study designs, are warranted. Overall, we highlight the importance of appraising known causal biomarkers before any causal interpretations of observational dietary data.

Food and nutrient intakes are largely influenced by environmental factors with minimal or no genetic contribution. However, when it comes to dietary behaviours, the role of genetics is plausible.^8^ Due to the inherent complexity related to the interplay between diet, genes and the environment, it is challenging to perform robust univariable Mendelian randomization analyses (Figure 1) to assess potential causality of primary dietary factors, including dietary behaviours. However, turning towards mediation analyses in the new framework of multivariable Mendelian randomization (Figure 2) appears to offer a fair amount of promise for assessing unknown potentially causal relations, mediation and pathways. In addition to strengthening overall aetiological understanding, this approach could be highly valuable in determining which RCTs would be the most likely to yield useful information.

## Funding

PO is supported by the Emil Aaltonen Foundation. MAK is supported by a research grant from the Sigrid Juselius Foundation, Finland.
